# Shelf life stability comparison in air for solution processed pristine PDPP3T polymer and doped spiro-OMeTAD as hole transport layer for perovskite solar cell

**DOI:** 10.1016/j.dib.2016.02.021

**Published:** 2016-02-15

**Authors:** Ashish Dubey, Nirmal Adhikari, Swaminathan Venkatesan, Shaopeng Gu, Devendra Khatiwada, Qi Wang, Lal Mohammad, Mukesh Kumar, Qiquan Qiao

**Affiliations:** a Center for Advanced Photovoltaics, Department of Electrical Engineering and Computer Science, South Dakota State University, Brookings, SD 57007, USA; b Functional and Renewable Energy Materials Laboratory, Department of Physics, Indian Institute of Technology Ropar, Punjab 140 001, India

**Keywords:** Pristine polymer, Hole transport layer, Perovskite, PDPP3T, Slower degradation

## Abstract

This data in brief includes forward and reverse scanned current density–voltage (*J*–*V*) characteristics of perovskite solar cells with PDPP3T and spiro-OMeTAD as HTL, stability testing conditions of perovskite solar cell shelf life in air for both PDPP3T and spiro-OMeTAD as HTL as per the description in Ref. [1], and individual *J*–*V* performance parameters acquired with increasing time exposed in ambient air are shown for both type of devices using PDPP3T and spiro-OMeTAD as HTL. The data collected in this study compares the device stability with time for both PDPP3T and spiro-OMeTAD based perovskite solar cells and is directly related to our research article “solution processed pristine PDPP3T polymer as hole transport layer for efficient perovskite solar cells with slower degradation” [2].

## Specifications Table

**Table:** 

Subject area	Physics
More specific subject area	Photovoltaics
Type of data	Table
How data was acquired	Solar Simulator (Xenon lamp-Newport), Agilent semiconductor parameter analyzer 4155C, Springfield precise temp humidity meter
Data format	Analyzed
Experimental factors	Current density-voltage (J-V) scans of perovskite solar cells with pristine PDPP3T and spiro-OMeTAD based HTL were immediately taken after fabrication. Same cells for stability measurement were used by keeping them in ambient air having 40% RH and scanning at regular interval.
Experimental features	J-V scans were carried out in ambient air by illuminating from the bottom electrode FTO contact.
Data source location	Department of Electrical Engineering and Computer Science, South Dakota State University, Brookings, SD
Data accessibility	Data is with this article.

## Value of the data

• Forward and reverse scanned current density–voltage (*J*–*V*) characteristics of perovskite solar cells with PDPP3T and spiro-OMeTAD as HTL can be used to find solar cell performance and demonstrate that PDPP3T is an effective dopant-free HTL. These data can provide guidance to other researchers that conduct similar research.
• Stability testing conditions and perovskite solar cell shelf life in air for both PDPP3T and spiro-OMeTAD as HTL can be used to study lifetime and repeatability measurements [1].
• Individual *J*–*V* performance parameters acquired with increasing time exposed in ambient air for both PDPP3T and spiro-OMeTAD based devices can be used to find which HTL leads to longer stability and lower degradation.

## 1 Data, experimental design, materials and methods

The data here provide device photovoltaic parameters and ambient air stability comparison for two different hole transport layers (PDPP3T and spiro-OMeTAD) based perovskite solar cells. Perovskite solar cells were fabricated with device structure as FTO/compact-TiO_2_/mesoporous-TiO_2_/Perovskite/HTL/Ag. Two different HTLs were used namely pristine polymer poly(diketopyrrolopyrrole-terthiophene) (PDPP3T) and doped small molecule 2,2′,7,7′-tetrakis(N,N-di-p-methoxyphenylamine)-9,9-spirobifluorene (spiro-OMeTAD). In our study, we have compared the device performance of perovskite solar cells with PDPP3T and spiro-OMeTAD based HTL. The devices were tested for their stability under the condition mentioned in Table 3. Solar cells were taken out of evaporator in ambient air immediately after fabrication for efficiency testing. We report various data accumulated from Perovskite layer XRD spectrum, *J*–*V* scans at different interval of time to monitor the ambient air stability for both PDPP3T and spiro-OMeTAD based perovskite devices.

Perovskite films were made using a two-step sequential deposition method and were characterized with X-ray diffraction to determine the crystalline perovskite phase. Full width half maxima (FWHM) of perovskite films determine the crystallinity of perovskite phase. Table 1 shows the FWHM of each characteristic peak of Perovskite (CH_3_NH_3_PbI_3_) phase.

Perovskite solar cells fabricated using PDPP3T and spiro-OMeTAD**-**based HTL were characterized for their *J*–*V* scans in both forward and reverse scan, immediately after evaporation of top silver electrode. The illuminated *J*–*V* scans in both forward and reverse scans were recorded and individual device parameters were calculated as shown in Table 2. All *J*–*V* curves (forward and reverse scan) were recorded with a scan rate of 1 V/s, with voltage step of 10 mV.

Table 3 shows detailed overview of conditions for test setup, testing protocols, output and equipment used for measuring the cells for air stability measurement. The measurement details described in Table 3 is as per testing protocols mentioned in reference [1].

Table 4 presents device performance parameters recorded for stability testing in air for both PDPP3T and spiro-OMeTAD based devices. *J*–*V* curves for all scans were recorded with a scan rate of 1 V/s, with voltage step of 10 mV. Fresh devices were fabricated and were immediately scanned to obtain *J*–*V* curves. The cells were then stored in a drawer and taken out to re-measure approximately after each day to see the performance levels for both PDPP3T and spiro-OMeTAD based perovskite solar cells.

## Figures and Tables

**Table 1:** Full width at half maximum (FWHM) at peaks at 14.03°, 28.36°, and 31.77 °.

Films	FWHM at 14.03° peak	FWHM at 28.36° peak	FWHM at 31.77° peak
TiO_2_+CH_3_NH_3_PbI_3_	0.378°	0.457°	0.476°

**Table 2:** Variation in device parameter in different cells in forward and reverse scan direction, for both PDPP3T and spiro-OMeTAD based cells.

Devices with	Scan direction	*J_sc_* (mA/cm^2^)	*V_oc_* (V)	FF (%)	Eff. (%)
PDPP3T as HTL	1-Forward	18.98	0.95	45.20	8.16
	1-Reverse	18.12	0.95	63.70	10.98
	2-Forward	19.62	0.96	47.80	9.01
	2-Reverse	20.52	0.98	61.25	12.32
	3-Forward	19.9	0.94	52.10	9.75
	3-Reverse	19.5	0.97	62.80	11.89
Spiro-OMeTAD as HTL	1′-Forward	22.57	0.87	55.90	10.99
	1′-Reverse	22.54	0.88	62.20	12.34
	2′-Forward	22.65	0.90	57.13	11.64
	2′-Reverse	22.82	0.89	58.98	11.98
	3′-Forward	20.09	0.90	53.10	9.60
	3′-Reverse	20.24	0.83	52.47	8.81

**Table 3:** Overview of organic–inorganic perovskite stability testing.

		**ISOS (D−1) shelf time**
Test setup	Light setup	Dark
	Load	Open circuit
	Storage temperature	Ambient (28 °C)
	Storage R.H.	Ambient (40% RH)
	Characterization light source	Solar Simulator (Xenon lamp)
Testing protocol	Storage temp./ R.H.	28 °C/40% RH
	JV characterization	In the range of 0 to 1 V
	Min. measurement intervals	Daily (approximately every 24.5 h)
	Characterization temperature	35 °C
	Characterization irradiance level	100 mW/cm^2^
Output	Time/date	Time interval for consecutive measurement is shown in Table 4.
	Characterization light source	Xenon lamp
	Storage temp./R.H.	25 °C/40% RH
	Instantaneous performance parameters	*J_sc_*, *V_oc_*, FF% and *ƞ*%
		*J_sc_*, *V_oc_*, FF% and *ƞ*% after each interval of time
	Stability performance parameters	
	Description of measurement protocol and testing setup	All measurement were done in ambient air. The cells were stored in a ambient air in drawer after each measurement and taken out only when re-measured after each day.
Required equipment	Characterization light source	Xenon lamp
	Temperature monitoring	Springfield Precise Temp humidity meter
	Humidity monitoring	Springfield Precise Temp humidity meter
	JV characterization	Solar simulator, Semiconductor parameter analyzer (Agilent 4155C)
	Storage	Drawer

**Table 4:** Device performance parameter with time for both PDPP3T and spiro-OMeTAD based perovskite solar cell with increasing storage time in ambient air.

Devices with	Time (h)	*J_sc_* (mA/cm^2^)	*V_oc_* (V)	FF (%)	Eff. (%)
PDPP3T as HTL	0	20.52	0.98	61.25	12.32
	26	22.2	0.89	51.7	10.23
	51	21.37	0.89	48.5	9.23
	73	20.9	0.89	48.6	9.04
	97	20.86	0.86	42.6	7.65
	124	19.91	0.73	41.6	6.05
	147	19.61	0.85	38.8	6.48
	172	19.88	0.6	40.6	4.85
Spiro-OMeTAD as HTL	0	22.54	0.88	62.2	12.34
	26	22.89	0.86	52.57	10.35
	51	21.67	0.83	45.02	8.09
	73	20.21	0.75	47.7	7.23
	97	17.9	0.75	34.43	4.62
	124	16.22	0.6	34.05	3.32
	147	13.78	0.63	35.04	3.04
	172	13.26	0.44	36.17	2.11
